# Analysis of the Distribution of Urine Color and Its Relationship With Urine Dry Chemical Parameters Among College Students in Beijing, China – A Cross-Sectional Study

**DOI:** 10.3389/fnut.2021.719260

**Published:** 2021-10-04

**Authors:** Jingnan Liu, Zijuan Zhang, Xiaohan Pang, Yaxing Cheng, Da Man, Xinyi He, Huihui Zhao, Ruizhen Zhao, Wei Wang

**Affiliations:** ^1^School of Traditional Chinese Medicine, Beijing University of Chinese Medicine, Beijing, China; ^2^Institute of National Medicine, Beijing University of Chinese Medicine, Beijing, China; ^3^The Third Affiliated Hospital of Beijing University of Chinese Medicine, Beijing, China

**Keywords:** urine color, urine specific gravity, CIE L^*^a^*^b^*^ color space, urine dry chemical analysis, hydration, chemical parameters

## Abstract

**Objectives:** The objective of this study was to provide a new classification method by analyzing the relationship between urine color (Ucol) distribution and urine dry chemical parameters based on image digital processing. Furthermore, this study aimed to assess the reliability of Ucol to evaluate the states of body hydration and health.

**Methods:** A cross-sectional study among 525 college students, aged 17–23 years old, of which 59 were men and 466 were women, was conducted. Urine samples were obtained during physical examinations and 524 of them were considered valid, including 87 normal samples and 437 abnormal dry chemistry parameters samples. The urinalysis included both micro- and macro-levels, in which the CIE L^*^a^*^b^*^ values and routine urine chemical examination were performed through digital imaging colorimetry and a urine chemical analyzer, respectively.

**Results:** The results showed that L^*^ (53.49 vs. 56.69) in the abnormal urine dry chemistry group was lower than the normal group, while b^*^ (37.39 vs. 33.80) was greater. Urine color can be initially classified based on shade by grouping b^*^. Abnormal urine dry chemical parameter samples were distributed more in the dark-colored group. Urine dry chemical parameters were closely related to Ucol. Urine specific gravity (USG), protein, urobilinogen, bilirubin, occult blood, ketone body, pH, and the number of abnormal dry chemical parameters were all correlated with Ucol CIE L^*^a^*^b^*^; according to a stepwise regression analysis, it was determined that more than 50% of the variation in the three-color space values came from the urine dry chemical parameters, and the b^*^ value was most affected by USG (standardized coefficient β = 0.734, *p* < 0.05). Based on a receiver operating characteristic curve (ROC) analysis, Ucol ≥ 4 provided moderate sensitivity and good specificity (AUC = 0.892) for the detection of USG ≥ 1.020.

**Conclusions:** Our findings on the Ucol analysis showed that grouping Ucol based on b^*^ value is an objective, simple, and practical method. At the same time, the results suggested that digital imaging colorimetry for Ucol quantification is a potential method for evaluating body hydration and, potentially, health.

## Introduction

Urine is a body fluid widely applied in biological and clinical research (1) and is endowed with easy and non-invasive sampling, with its measurement value being relatively stable (2). A urinalysis includes both macroscopic and microscopic parts. On the microscopic side, a urinalysis is a powerful tool for collecting important diagnostic information in medicine (3). The chemical examination of urine includes the identification of protein, blood cells, glucose, pH, bilirubin, urobilinogen, ketone body, nitrite, and white blood cells esterase (3, 4). Urine can also sensitively detect the pathological alterations that occur in various diseases (5), such as bladder and prostate cancer (6, 7). In contrast, the macroscopic aspects refer to the color and cloudiness analyses (8, 9). Indeed, the urinalysis for medical purposes could date back to ancient Egypt, where urological examinations identified various diseases by examining the color, cloudiness, smell, and even taste of urine. Specifically, brown urine indicates jaundice, red (blood) urine indicates urinary tract tumors, colorless urine indicates diabetes mellitus, and foamy urine indicates proteinuria (4). In 2008, the urine diagnosis practiced in Chinese Tibetan medicine has been included in the first batch of the national intangible cultural heritage expansion project list of China (10). By identifying the macroscopic characteristics and changes of urine, the location, nature, and outcome of a disease can be judged, which can be used for its diagnosis and differential diagnosis, its formulation of treatment principles, and it prompting of treatment contraindications (11, 12).

Compared with microscopic tests, urine color (Ucol) is more accessible and easier to observe. Therefore, we further explored in the current study how these two correlate, as we sought to reflect microscopic sides from a macroscopic view. Macroscopic color alterations in urine are definitely the result of microscopic changes. Urine color depends on the concentration of urine pigment produced by hemoglobin catabolism (4, 13). Urine color has also been proved to be related to hydration status in healthy adults and is significantly related to two defined physiological parameters of hydration, namely, urine osmolality and urine specific gravity (USG) (14–17). However, the relationship between Ucol and other microscopic parameters is also important, which makes it necessary to explore the relationship between Ucol and urine dry chemical parameters.

Most studies used an eight-point color scale by Armstrong (14, 18) to subjectively evaluate Ucol. The scale ranged from light yellow to dark amber to analyze the correlation of Ucol with hydration (15–17, 19–21). It is an effective method that is highly practical for assessing hydration either for researchers or self-assessment (17, 22, 23). However, this subjective tool has certain methodological limitations, including its lack of objective quantification (24) and the limited content in its interval scale, as it is without continuous numerical value [Color 1, 255255199; Color 2, 255251186; Color 3, 255249164; Color 4, 255232077; Color 5, 245227157; Color 6: 229201135; Color 7, 207159072; Color 8, 147140074 (RGB, Södersjukhuset Photographic Laboratory, Stockholm, Sweden)] (20). Nonetheless, it is also important to enhance its practicality through other methods based on this scale. The advent of the Internet era has brought new ways of life to humans, and the trend is prominent in the utilization of smartphones to monitor health conditions. Chin et al. (25) designed an Internet of Things (IoT)-based pervasive body hydration tracker, within which external components, such as sensors, measure Ucol and upload to cell phones to infer hydration status by analyzing the B value of Ucol RGB; however, the device was much too complex. Instead, cameras and computers allow for the fast processing of images in terms of color balance and compensation. The digital imaging colorimetric method uses digital devices to record colors and numerically represent their shades. Chew et al. (26) verified that digital imaging colorimetry using cell phones was highly correlated with the hydration status of dengue patients by analyzing the correlation between RGB values and established laboratory parameters of urine photographs taken by Photoshop extraction cell phones. In addition, no other studies on Ucol distribution have been seen.

The International Commission on illumination (CIE) recommended an objective tristimulus colorimetry, which is the CIE L^*^ a^*^ b^*^. It is based on the perception of color and is independent of light and equipment. It has been used for the objective analysis of color and its movement, such as studying the color distribution of human gingiva (27, 28). In this system, the specific location of color space is defined by three coordinates, namely, L^*^, a^*^, and b^*^, with L^*^ being the luminance part and a^*^ and b^*^ being the color parts. The larger value of L^*^ indicates higher luminance, the negative values of a^*^ are presented as green, and the positive value is red. The negative values of b^*^ are presented as blue while the positive value is yellow. In addition, CIE L^*^ a^*^ b^*^ can calculate the difference between two colors. At present, it is recommended to use the CIEDE2000 color difference (ΔE) formula (27). Previous studies have used CIE L^*^a^*^b^*^ to identify Ucol, illustrating that the difference in Ucol regarding hydrated status is related to urine osmolality (29, 30).

Considering this, our study aimed to take urine images and obtain CIE L^*^ a^*^ b^*^ values with specific filming equipment. Firstly, we analyzed the quantitative differences in Ucol between normal and abnormal samples of urine dry chemistry parameters. Secondly, we used a new Ucol classification method to initially explore the distribution of Ucol. Next, we analyzed the relationship between Ucol and urine dry chemistry parameters. Finally, the accuracy of Ucol for assessing hydration was determined. From an objective perspective, we explored the distribution of Ucol and the influence of urine dry chemical parameters on it, facilitating the objectification and modern application of the traditional medical urological diagnosis.

## Materials and Methods

### Participants

A half-month cross-sectional study was conducted on newly enrolled students for a physical examination in a university in Beijing in September. The study protocol was approved by the Ethics Review Committee of Beijing University of Traditional Chinese Medicine (approval number: 2020BZYLL0301) and was conducted according to the guidelines of the Declaration of Helsinki.

Urine samples were collected during two batches of physical examination. A total of 525 participants were recruited in the study, and one sample with abnormal images was excluded. Among them, 87 samples were normal regarding urine dry chemical parameters and 437 samples were abnormal.

The two batches of urine samples were collected at different times. Since we only wanted to understand the distribution of abnormal samples in the early-stage study, we randomly kept part of the abnormal urine dry chemistry parameter samples. In the later stage of our study, we conceived a comparison study, so all samples were kept for the second time.

The study collected sample for the first time was from 7:00 to 10:00 a.m. There were 253 samples, of which 59 came from men and 195 came from women, with an average age of 20.50 ± 3.04. The imbalance in the ratio of men to women students in this school was one of the reasons for the imbalance in samples. The second time was from 8:30 to 10:00 a.m. There were 272 samples, and because of the need to avoid the menstrual cycle, the samples from women students had an average age of 18.4 ± 1.81. There were 88 samples with normal urine dry chemical parameters (one sample was excluded due to an abnormal image) and 184 samples with abnormal parameters. Diet and vitamin intake were not controlled and female participants were out of their menstrual cycles.

### Study Procedure

On the day of the study, participants first collected their urine. Afterward, the investigators tested the urine dry chemical parameters, then the investigators obtained urine images and analyzed the images accordingly. The entire process was completed within 2 h, and the process was discussed in the following paragraphs.

Urine collection: In the laboratory toilet, participants collected their mid-portion urine using a disposable urine cup and then transferred a part of the urine to a hospital-standard 10-ml disposable urine collection tube. The average transferred volume of urine was 8–10 ml. After the collection, they immediately handed over the urine sample to the investigators.

Dry chemical analysis of urine: A total of 11 parameters were obtained by professional testers in the lab using a Deere H-800 urine analyzer (Deere & Company, Changchun, China), and the instrument was calibrated using quality control samples before testing. Urine samples were tested without any modifications or alterations.

**Figure 1:**
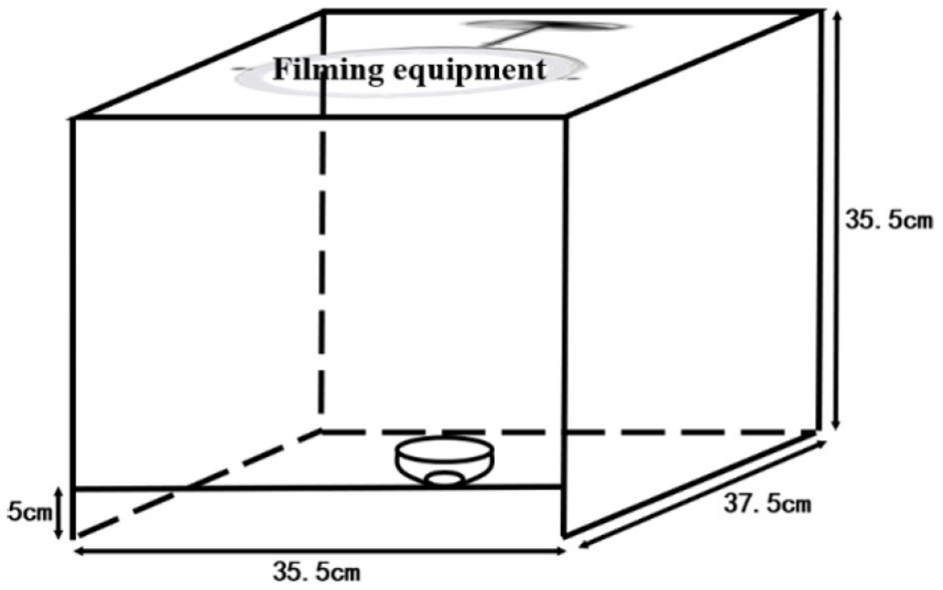
The urine image device.

Urine color collection method: The images of urine samples were taken immediately after the measurement of urine dry chemical parameters. The urine from the collection tube was transferred to a white porcelain bowl by the investigators. The bowl was marked by a measuring cylinder with 8 ml, and the urine was added to 8 ml; thus, the urine volume was roughly quantified as 8 ml. The urine image device is shown in Figure 1. To eliminate the effects of light and other environmental factors, we used a custom-made filming box (35.5 × 37.5 × 35.5 cm) with openings at the top and bottom and another 5-m opening directly in front of the box. The box was made of wood with a white interior. A Laikang mirror (Laikang Technology Co., Beijing, China) was placed on the top and then covered above to ensure a closed environment. At the bottom, we placed a sheet of white paper. We photographed it with a Laikang mirror with its own light source (color temperature 5500K, white light, 0–255 brightness adjustment) and 13 million px. A white porcelain bowl containing a quantitative amount of urine was placed under the lens of the Laikang mirror and a color correction color card CASMATCH (Bear Corporation, Tokyo, Japan) was placed next to it. Then, the jpeg format of each image was obtained by connecting the Laikang mirror to Bluetooth and using a remote control in a cell phone for taking pictures. Urine color was analyzed using the Adobe Photoshop CS5 (Adobe Systems, USA) software for urine images, and a fixed size (300 × 300 px) area close to the bottom of the cup size was selected by the ellipse selection tool, while the average CIE L^*^a^*^b^*^ values for each fixed area of the image were obtained using the software filter-blur-average-collection (26).

### Definition of Normal and Abnormal Urine

The samples were divided into normal and abnormal groups according to the analyzer results. As long as one parameter was abnormal, the sample would be considered abnormal. Samples were only regarded as normal when all the parameters were in normal reference values.

### Grouping Criteria of Ucol

The grouping criteria were determined according to five main aspects: ① the color of the normal urine ranges from light yellow to dark amber depending on the content of urine pigment, while it is all yellow; ② previous studies (31, 32) demonstrated the significant relationship between the b^*^ value of the CIE L^*^a^*^b^*^ color space and hydration status (numerical grade of the eight-point color scale); ③ the value with a wide range span was selected according to the actual situation of the samples in order to make a good classification [in the normal group of samples, the b^*^ value (11–52) had the largest span compared to the L^*^ value (43 ~ 68) and the a^*^ value (2 ~20)]; ④ there should be a certain color difference between adjacent groups so that the human eye can distinguish between them, since excessive groups would be hard to distinguish and too few groups would not be very meaningful; ⑤ it should be objective, simple, and convenient in application. Therefore, Ucol was classified according to the method of grouping mainly by the b^*^ value, and if necessary, other values were considered secondarily.

Firstly, the samples were classified based on the b^*^ value. The range of b^*^ values for normal samples was 11–52. The range of the b^*^ values for the abnormal samples was 12–60 after excluding one sample with a black color (b^*^ = 0). The intergroup spacing was set to 10 and divided into five groups. Some samples were in a special location in the CIE L^*^a^*^b^*^ three-dimensional color space, and their a^*^ values were observed to be higher (redder component). Hence, we defined their a^*^ range. and the range of this group was initially determined to make it a group alone. The darkest color was observed with the human eye in this group, so it was set as the sixth group. The rest of the groups were arranged in ascending order based on the b^*^ value, making Ucol grouped from light to dark. The first five groups required only one dimension of b^*^, while the sixth group required consideration of both the b^*^ and a^*^ dimensions. The grouping criteria for the six groups of Ucol are shown in Table 1.

**Table 1:** Grouping criteria of urine color.

	**Group 1**	**Group 2**	**Group 3**	**Group 4**	**Group 5**	**Group 6**
**Urine color**						
b*	11 ~ 20	21 ~ 30	31 ~ 40	41 ~ 50	51 ~ 60	34 ~ 50
a*						20 ~ 38

### Statistical Analysis

The data analysis was performed on IBM SPSS Statistics version 20 (IBM Co., USA). The quantitative parameters of participants were expressed as mean ± SD. First, the mean values of both normal and abnormal urine CIE L^*^a^*^b^*^ from the second batch of samples were calculated, and the difference in the Ucol space values between the abnormal and normal urine dry chemistry groups was determined by a nonparametric test.

Next, the color distribution of all urine samples in the CIE L^*^ a^*^ b^*^ color space was observed. The distribution of the abnormal samples of the second batch was analyzed in detail. The samples were grouped according to the criteria, and then the percentage of abnormal samples in each group was calculated to analyze the color distribution of the abnormal group. The color difference calculation was implemented by an Excel spreadsheet with a parameter factor set to one provided by Sharma et al. (33).

Then, a Spearman correlation analysis was performed to determine the correlation between Ucol and urine dry chemical parameters. The value of |r| ≤ 0.3 was considered a weak correlation, 0.3 < |r| 6 was considered as moderate correlation,0.6 < |r| ≤ 0.8 was considered a strong correlation, and |r| > 0.8 was considered a very strong correlation. Based on the urine dry chemical parameters, a stepwise regression analysis was used to predict the color characteristics of urine. The closer the coefficient of determination *R*^2^ was to 1, the better the fit of the regression equation was.

**Figure 2:**
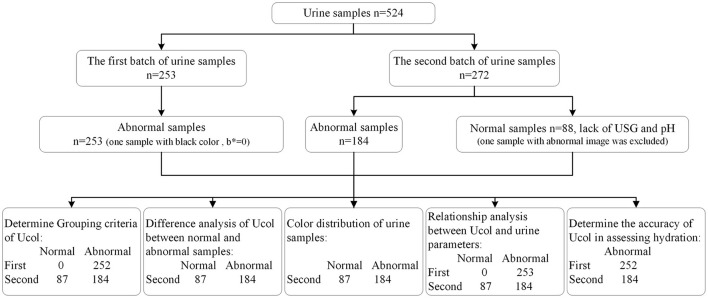
Consort diagram for the study.

Lastly, the ability of this new Ucol classification method to assess hydration was determined. A receiver operating characteristic curve (ROC) was generated using the Ucol predictor variable, matching USG ≥ 1.020 as the urine concentration threshold (33, 34). To determine the optimal cutoff value for Ucol used to identify USG ≥ 1.020, the maximal approach of the sensitivity and specificity was used. When interpreting the area under the curve (AUC) a value of ≥ 0.90 as excellent, 0.80–0.89 was considered good and 0.70–0.79 was considered fair. The probability (p) level of 0.05 was defined as statistically significant. The consort diagram for the study is shown in Figure 2.

## Results

### The Differences in Ucol Between Urine Dry Chemistry Parameters Normal and Abnormal Group

The L^*^ value of the abnormal group was significantly lower than that of the normal group (53.49 vs. 56.69); the difference in a^*^ value between the two groups was not statistically significant (10.31 vs. 8.69); the b^*^ value of the abnormal group was significantly higher than that of the normal group (37.39 vs. 33.80) (Table 2).

**Table 2:** Comparison of urine samples with normal and abnormal urine dry chemical parameters in CIE L^*^a^*^b^*^.

	**All urine samples** **(*n* = 271)**	**Normal group** **(*n* = 87)**	**Abnormal group** **(*n* = 184)**	**Z**	***p***
**Urine color**					
L^*^	54.52 ± 5.50	56.69 ± 5.27	53.49 ± 5.32	−4.06	0.000^*^
a^*^	9.79 ± 5.34	8.69 ± 4.12	10.31 ± 5.77	−1.80	0.072
b^*^	36.24 ± 11.27	33.80 ± 10.97	37.39 ± 11.25	−2.47	0.014^*^

*All values were shown as means ± SD*.

* *There was a statistically significant difference between the normal group and the abnormal group, p < 0.05*.

### The Color Distribution of Urine Samples

**Figure 3:**
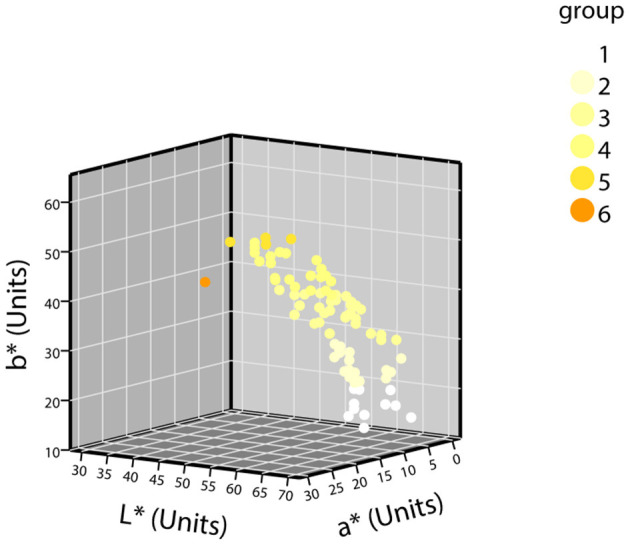
The distribution of CIE L*a*b* values of normal urine samples in a three-dimensional space (*n* = 87).

**Figure 4:**
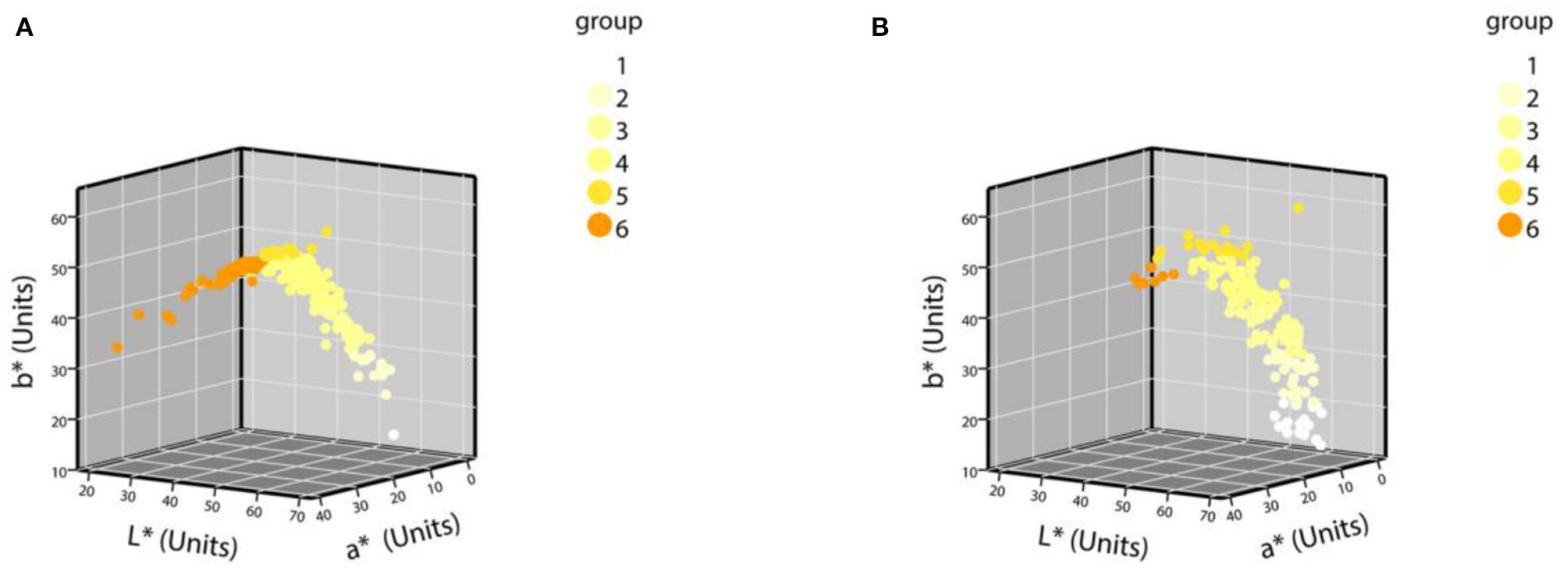
The distribution of CIE L^*^a^*^b^*^ values of abnormal urine samples in a three-dimensional space. **(A)** The first batch of urine samples with abnormal urine dry chemical parameters (*n* = 252); **(B)** the second batch of urine samples with abnormal urine dry chemical parameters (*n* = 184).

The samples were grouped according to the grouping criteria. The distribution of the CIE L^*^a^*^b^*^ values of the normal urine samples in the three-dimensional space is shown in Figure 3. The first batch grouping distribution of the CIE L^*^a^*^b^*^ values of the abnormal urine samples in the three-dimensional space is shown in Figure 4A (one black sample with b^*^ = 0 was excluded), with the second batch shown in Figure 4B.

The detailed distribution of the second batch of urine samples was analyzed. The grouping results of urine samples with normal-dry chemical parameters are shown in Table 3. Of the normal samples, 98.85% were distributed in groups 1–5, and the sixth group with the darkest color was rarely distributed. The results of the grouping of samples with abnormal parameters and the percentage of abnormal groups are shown in Table 4. The percentage of abnormal parameters in groups 4–6 with darker colors were all higher and all exceeded the average percentage.

**Table 3:** Grouping results of urine samples with normal urine dry chemical parameters (*n* = 87).

	**Group 1**	**Group 2**	**Group 3**	**Group 4**	**Group 5**	**Group 6**
L*	59.25 ± 4.49	58.47 ± 3.99	58.03 ± 4.79	53.43 ± 4.71	51.00 ± 3.74	43.00 ± 0.00
a*	3.08 ± 0.79	5.42 ± 1.17	8.67 ± 1.42	12.86 ± 1.59	16.50 ± 1.29	20.00 ± 0.00
b*	15.58 ± 3	24.89 ± 2.71	36.20 ± 3.03	45.05 ± 3.06	51.50 ± 0.58	43.00 ± 0.00
N	12	19	30	21	4	1

*The color difference of the color means between the two adjacent groups of the normal samples was 3.45~9.18 units*.

**Table 4:** Grouping and percentage of urine samples with abnormal urine dry chemical parameters (*n* = 271).

**Group**	**Group 1**	**Group 2**	**Group 3**	**Group 4**	**Group 5**	**Group 6**	**Total**
Number of normal samples	12	19	30	21	4	1	87
Number of abnormal samples	19	33	47	59	16	10	184
Total	31	52	77	80	20	11	271
Percentage of abnormal samples	61.29%	63.46%	61.04%	73.75%	80.00%	90.91%	67.90%

### Relationship Between Ucol and Urine Dry Chemical Parameters

**Figure 5:**
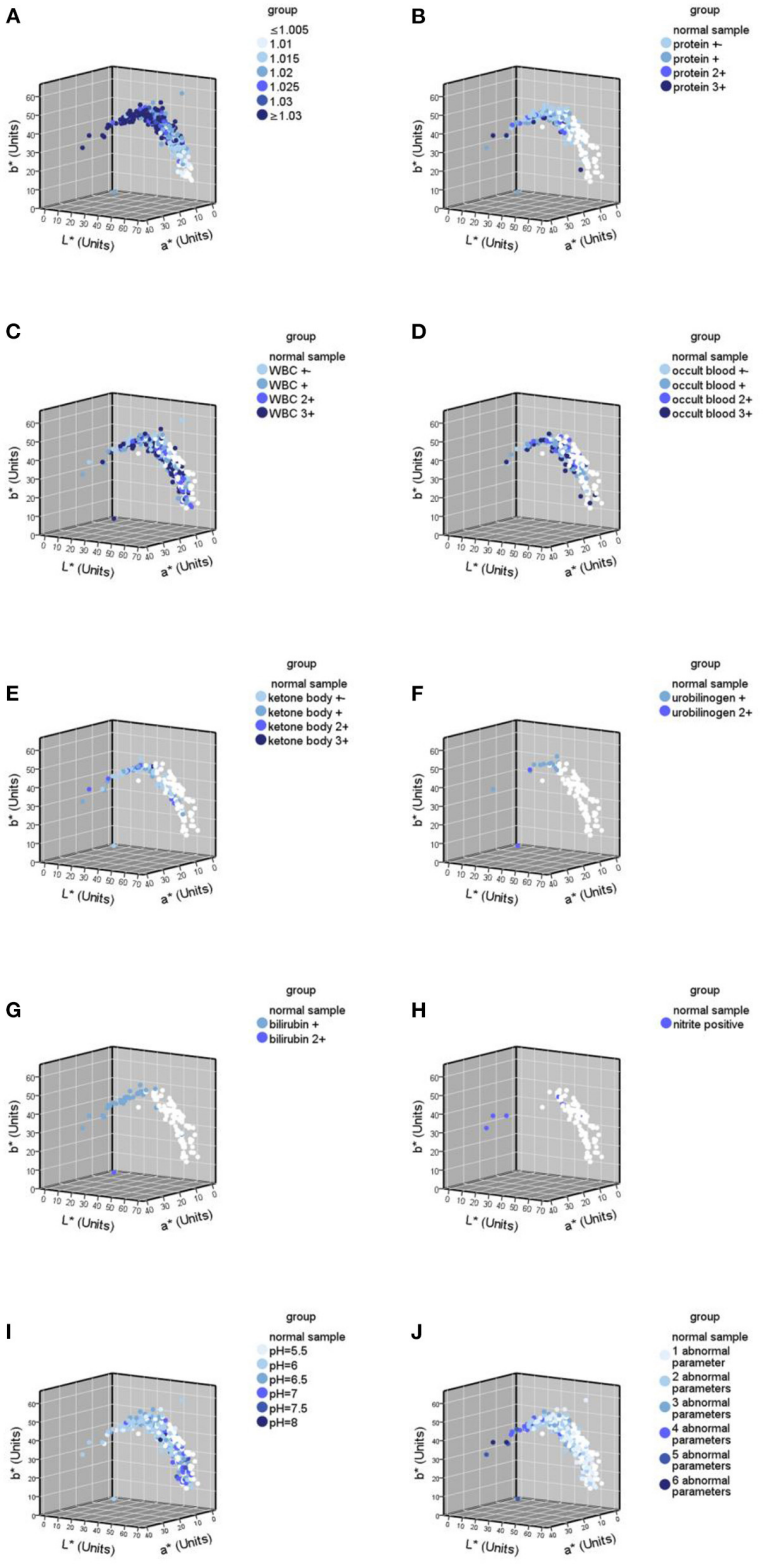
Urine color distribution diagram of various abnormal parameters in urine samples (*n* = 524). **(A)** Urine color distribution diagram classified by urine specific gravity (USG); **(B–H)** urine color distribution diagram of various parameters; **(B)** yhe diagram of urine samples containing protein abnormalities; **(C)** the diagram of urine samples containing white blood cell (WBC) abnormalities; **(D)** the diagram of urine samples containing occult blood abnormalities; **(E)** the diagram of urine samples containing ketone body abnormalities; **(F)** the diagram of urine samples containing urobilinogen abnormalities; **(G)** the diagram of urine samples containing bilirubin abnormalities; **(H)** the diagram of urine samples containing nitrite abnormalities; **(I)** the diagram classified by pH; **(J)** the diagram classified by the number of abnormal parameters.

The USG and pH information were missing in the normal samples, so only abnormal urine samples (437) were involved in the analysis of the correlation between urine specific gravity/pH and urine color, while all samples (524) were involved in the analyses of other remaining parameters. The results of the Spearman correlation analysis (Table 5) showed that USG was significantly strongly correlated with CIE L^*^a^*^b^*^ values, negatively correlated with L^*^ (*r* = −0.620, *p* < 0.05), and positively correlated with a^*^ (*r* = 0.664, *p* < 0.05) and b^*^ (*r* = 0.614, *p* < 0.05). Protein was strongly negatively correlated with L^*^ (*r* = −0.605, *p* < 0.05), strongly positively correlated with a^*^ (*r* = 0.627, *p* < 0.05), and moderately positively correlated with b ^*^ (*r* = 0.539, *p* < 0.05). The number of abnormal urine dry chemical parameters was moderately correlated with all three values, negatively correlated with L^*^ (*r* = −0.578, *p* < 0.05), and positively correlated with the a ^*^ (*r* = 0.495, *p* < 0.05) and b^*^ values (*r* = 0.401, *p* < 0.05). Bilirubin and ketone body were moderately negatively correlated with L^*^ (*r* = −0.368, −0.316, both *p* < 0.05) and positively correlated with a^*^ (*r* = 0.369, 0.242, both *p* < 0.05) and b^*^(*r* = 0.189, 0.162, both *p* < 0.05), respectively. pH had a significant moderately positive correlation with L^*^ (*r* = 0.358, *p* < 0.05) and a significant negative correlation with a^*^ (*r* = −0.345, *p* < 0.05) and b^*^ (*r* = −0.257, *p* < 0.05). Urobilinogen had a significant negative weak correlation with L^*^ (*r* = −0.154, *p* < 0.05) and a significant weak positive correlation with a^*^ (*r* = 0.168, *p* < 0.05) and b^*^ (*r* = 0.189, *p* < 0.05). Occult blood had a significant weak negative correlation with the L^*^ value (*r* = −0.194, *p* < 0.05), a significant weak positive correlation with a^*^ (*r* = 0.095, *p* < 0.05), and no significant correlation with b^*^. There was no significant correlation between white blood cells and nitrite with CIE L^*^a^*^b^*^ values. The urine color distribution diagram of various parameters is shown in Figure 5, which intuitively shows this conclusion.

**Table 5:** Spearman correlation analysis results of urine color space values and urine dry chemical parameters.

	L^*^ **rs (p)**	a^*^ **rs (p)**	b^*^ **rs (p)**
USG	−0.620^**^ (0.000)	0.664^**^ (0.000)	0.614^**^ (0.000)
Protein	−0.605^**^ (0.000)	0.627^**^ (0.000)	0.539^**^ (0.000)
White blood cells	−0.065 (0.140)	−0.057(0.194)	−0.075 (0.088)
Occult blood	−0.194^**^ (0.000)	0.095^*^ (0.030)	0.046 (0.295)
Ketone body	−0.316^**^ (0.000)	0.242^**^ (0.000)	0.162^**^ (0.000)
Urobilinogen	−0.154^**^ (0.000)	0.168^**^ (0.000)	0.189^**^ (0.000)
Bilirubin	−0.368^**^ (0.000)	0.369^**^ (0.000)	0.189^**^ (0.000)
Nitrite	−0.081 (0.064)	0.080 (0.068)	−0.021 (0.627)
pH	0.358^**^ (0.000)	−0.345^**^ (0.000)	−0.257^**^ (0.000)
The number of abnormal parameters	−0.578^**^ (0.000)	0.495^**^ (0.000)	0.401^**^ (0.000)

*rs denotes Spearman's rho coefficient*.

** *Denotes p-value < 0.01*.

* *Denotes p-value < 0.05*.

The results of the stepwise regression analysis of CIE L^*^a^*^b^*^ color space values and urine dry chemical parameters are shown in Table 6. The stepwise regression model had significance in predicting the space values of urine color (*p* < 0.05 for all three models, adjusted *R*^2^ = 0.559, 0.586, 0.527). Furthermore, the L^*^ value had the largest significant correlation with the urine dry chemical parameters (USG > protein > urobilinogen > the number of abnormal parameters > bilirubin > pH > ketone body, all *p* < 0.05). These seven variables explained 55.9% of the L^*^ values. The a^*^ value had the largest significant correlation with the urine dry chemical parameters (USG > the number of abnormal parameters > protein > occult blood > white blood cells > bilirubin > urobilinogen, all *p* < 0.05). These seven variables explained 58.6% of the a^*^ values. Urine specific gravity, a parameter related to hydration, had the highest effect on the three values. Urine specific gravity also had the greatest effect on b^*^ value (standard coefficient β = 0.734, *p* < 0.05). Five variables (USG > urobilinogen > protein > bilirubin > pH) could account for 52.7% of the b^*^ values. The adjusted *R*^2^ value was used to determine this percentage of variance, as this value was based on the sample size and the number of predictor values contributing to the regression model. The coefficients for each predictor value that contributed to the construction of the stepwise regression models have been reported (Table 6).

**Table 6:** Stepwise regression analysis results of urine color and urine dry chemical parameters (*n* = 437).

	**Non-standardized coefficient β**	**Standard coefficient β**	***t*-value**	***p*-value**	***F* value of model**	***p*-value of model**	***R* ^**2**^**	**adjusted *R*^**2**^**
L*								
Constant	61.702		22.164	0.000	79.950	0.000	0.566	0.559
USG	−1.220	−0.324	−7.928	0.000				
The number of abnormal parameters	−1.145	−0.158	−3.427	0.001				
Urobilinogen	−3.846	−0.213	−6.495	0.000				
Protein	−1.637	−0.240	−6.331	0.000				
Bilirubin	−1.861	−0.152	−3.791	0.000				
pH	0.934	0.094	2.517	0.012				
Ketone body	−0.736	−0.073	−1.968	0.050				
a*								
Constant	−0.743		−0.602	0.548	89.067	0.000	0.592	0.586
USG	1.578	0.451	12.608	0.000				
Bilirubin	1.382	0.121	2.907	0.004				
Protein	0.996	0.157	4.013	0.000				
Urobilinogen	1.843	0.110	3.378	0.001				
The number of abnormal parameters	1.685	0.249	4.768	0.000				
Occult blood	−0.635	−0.125	−3.360	0.001				
White blood cells	−0.514	−0.122	−3.207	0.001				
b*								
Constant	9.983		2.514	0.012	98.062	0.000	0.532	0.527
USG	3.825	0.734	17.472	0.000				
Urobilinogen	3.412	0.137	4.050	0.000				
pH	1.281	0.093	2.405	0.017				
Protein	1.063	0.113	2.987	0.003				
Bilirubin	−1.796	−0.106	−2.959	0.003				

### Accuracy of Ucol for Assessing Hydration

The ROC analysis revealed the optimal Ucol cutoff for identifying USG ≥ 1.020 was 4 (AUC = 0.892). A Ucol of ≥4 offered moderate sensitivity and good specificity (Table 7).

**Table 7:** Urine samples classified according to USG and urine color (Ucol) values; followed by metrics from the receiver operating characteristic curve (ROC) analysis.

	**Diagnostic Standard**		**Metrics from ROC analysis^a^**				
	**USG ≥ 1.020** **(*n* = 337)**	**USG <1.020** **(n = 99)**	**Sensitivity**	**Specificity**	**Accuracy**	**PPV**	**NPV**
Ucol ≥ 4	268	12	0.795	0.879	0.814	0.957	0.558
Ucol <4	69	87					

a *Sensitivity: percentage of true positives (that is, USG ≥ 1.020) detected by UCol ≥ 4. Specificity: percentage of true negatives (that is, USG <1.020) detected by Ucol <4. Accuracy: percentage of all samples (positive or negative) accurately classified by UCol. Positive predictive value (PPV): probability that a urine sample with UCol ≥ 4 has a USG ≥ 1.020. Negative predictive value (NPV): probability that a urine sample with Ucol <4 has a USG <1.020*.

## Discussion

Our study showed that the Ucol obtained from general filming equipment can be used in analytical studies. Abnormal urine samples were darker and more yellowish than the normal ones. Urine color could be classified based on shade mainly by grouping b^*^ value, and urine samples with abnormal dry urine chemistry parameters were more distributed in darker areas. Nine dry urine chemistry parameters, which are USG, protein, bilirubin, urobilinogen, white blood cells, ketone body, occult blood, pH, and number of abnormal parameters, were correlated with Ucol space values. This new method of Ucol grouping had good accuracy in the assessment of hydration. To our knowledge, this is one of the first studies to use quantitative values to classify Ucol.

Methodologically, to make the measurement easier, we adopted digital imaging colorimetry to record Ucol, which has the advantages of flexibility and convenience, rapidity, economy, objectivity, reproducibility, permanent storage, and accuracy. However, it can be affected by light, filming equipment, operator, and analysis software. As a consequence, the obtained chromaticity value will have a certain deviation (35, 36). The Laikang mirror came with its own light source, and the customized shooting box ensured a fixed height and angle for measurement. Photoshop was used to objectively quantify the average regional Ucol, maximizing control over the shooting environment and other conditions, to make the results accurate and objective. Since the urine images were taken in a unified background, no color cards were applied to eliminate background differences. The collected data were used to analyze the distribution of Ucol in CIE L^*^a^*^b^*^ and the relationship between Ucol space values and urine dry chemical parameters.

To the best of our knowledge, the current eight-point color scale developed by Armstrong et al. (14, 18) is an effective method for assessing the relationship between Ucol and hydration in healthy adults. Urine color has been shown to be closely related to hydration status in daily activities, exercise, hydropenia, or in children and women (8–14 years) (19–21, 37, 38). Meanwhile, the IoT-based pervasive body hydration tracker developed by Chin et al. (25) showed that the method of inferring the hydration by considering only the B value of RGB was correct. Drawing on the experience of the two methods above, we chose to classify the Ucol mainly by grouping the b^*^ value, and preliminarily identified six groups and determined the distribution of the most common Ucol. Hahn et al. (20) provided adapted RGB values (Södersjukhuset Photographic Laboratory, Stockholm, Sweden) based on the original Armstrong color scale, and we converted them to the CIE L^*^a^*^b^*^. We then found the fourth group of colors of this value that did not appear in our normal samples and only one case in our abnormal samples (Figure 4B). The one was not grouped separately due to the relatively small number, suggesting that we still need to expand our sample size. For grouping, the eight-point color scale was based on observations of urine samples, and was then printed on a laminated chart (18) with an uneven distribution between groups. We classified mainly by grouping the b^*^ value, with isometric grouping to make it simple and practical. The color difference between the means of each adjacent two groups after the grouping of normal samples was calculated and ranged from 3.45 to 9.18 units. For the eight-point color scale, it was 2.44–23.12 units. The study showed that the color difference equal to or beyond three could be well recognized by the human eye (39), which indicates that the grouping criteria had a certain distance between groups in practical application. In addition, due to methodological issues such as light sources and pixels, our color space values were low overall, and the equipment needs improvement.

The results of our study showed that, firstly, the differences in Ucol parameters were found between normal and abnormal urine dry chemistry parameter groups. Using the results of urine dry chemistry analysis, all samples were divided into normal and abnormal parameters groups. The nonparametric tests analyzing the CIE L^*^a^*^b^*^ values of the two groups showed that the L^*^ and b^*^ values differed between the two groups, such that the lower the L^*^ value, the lower the brightness. When the b^*^ value was positive, there was a positive correlation between the b^*^ value and the yellow component, showing that the abnormal group had darker and more yellow Ucol than the normal group (Table 2). For the first time, such an analysis was performed, suggesting that alterations to dry chemical parameters lead to changes in the color of urine, most of which are darker.

Secondly, the color distributions of samples with normal and abnormal parameters were different. In our second batch, 98.85% of the normal samples were distributed in groups 1–5, and only a small portion was distributed in the darkest color group. Abnormal samples accounted for a higher percentage in the darker groups 4–6 (Table 4), showing that the samples with abnormal urine parameters were distributed more in the darker areas. It was suggested that if the Ucol is consistently dark, there may be abnormalities in the body, and vice versa if the urine is consistently light, implying that there may be also abnormalities. Meanwhile, the concentration of urine, which is influenced by many parameters, also affects urine color (40), showing that urine color is related to both urine concentration and urine dry chemical parameters. Although for the second time we collected the entire samples without screening, the number of normal samples was relatively small compared with the abnormal ones. The results are yet to be validated with a larger sample size in the future.

Thirdly, the reasons for the color differences between normal and abnormal urine dry chemistry parameters were further analyzed. Correlation and stepwise regression analyses were performed. Several studies have shown a significant relationship between Ucol and osmolality as well as USG (14–17). Our study found a significant and strong correlation between USG and all three space values of Ucol (Table 5). They negatively correlated with the L^*^ value (*r* = −0.620, *p* < 0.05) and positively correlated with a^*^ (*r* = 0.664, *p* < 0.05) and b^*^(*r* = 0.614, *p* < 0.05), which indicated a higher USG, lower brightness, and a higher portion of the red and yellow components, namely, the darker Ucol. In assessing the effect of hydration on Ucol, belasco et al. (32) demonstrated a significant decrease in L^*^ value and a significant increase in b^*^ value with increasing urine osmolality, while a^*^ value was presented as a parabolic curve: decrease at first and then increase. Urine specific gravity and osmolality correlated strongly (*r* = 0.97) (31), as seen in agreement with the changes in the L^*^ and b^*^ values studied by Belasco et al. In the regression analysis, USG also had an effect on three color space values, with USG having the greatest effect on the b^*^ value (standardized coefficient β = 0.734, *p* < 0.05), which showed that USG had a strong effect on Ucol.

In addition, Ucol space values were also correlated with protein, urobilinogen, bilirubin, white blood cells, occult blood, ketone body, pH, and the number of abnormal urine dry chemical parameters, but not with nitrite. Protein was highly correlated with CIE L^*^a^*^b^*^ values and had a great effect on all three values. In the correlation analysis, abnormal samples of bilirubin and urobilinogen were fewer and were weakly correlated with CIE L^*^a^*^b^*^, while in the regression analysis, both were shown to affect CIE L^*^a^*^b^*^ values (Table 6), which indicated that these two parameters had a greater effect on Ucol. White blood cells were not correlated with urine CIE L^*^a^*^b^*^ values in the correlation analysis, but were shown to affect a^*^ values to a lesser extent in the regression analysis. Occult blood was weakly correlated with the L^*^ and a^*^ values in the correlation analysis, but was shown to affect only a^*^ in the regression analysis. Ketone body and pH correlated weakly with CIE L^*^a^*^b^*^ values, and ketone body had an effect on L^*^ and pH on a^*^ and b^*^ in the regression analysis. It can be seen that white blood cells, occult blood, ketone body, and pH have some degree of influence on Ucol, but this was relatively low. The number of abnormal urine dry chemical parameters, a parameter calculated at a later stage, was also correlated with Ucol, indicating that microscopic accumulation affected macroscopic color changes. This was the first study to parameterize the number of abnormal urine dry chemical parameters. Nitrite was not associated with CIE L^*^a^*^b^*^ values in both the correlation and regression analyses, indicating that it did not affect Ucol. Chew et al. (26) related urine RGB to urine chemical parameters with urine images, demonstrating that urine RGB values were also strongly related to urine chemical parameters, with all three RGB values showing significant negative correlations with urine osmolality and USG (all *r* < −0.701, *p* < 0.001) and significant correlations with urine protein, ketone body, bilirubin, urobilin, and urine hemoglobin. Although the color systems were different, all indicated a close relationship between Ucol and urine chemistry parameters.

The successful findings of the correlation analysis prompted the construction of regression models (Table 6), suggesting the predictive potential of urine dry chemical parameters for Ucol. In the stepwise regression analysis, the adjusted *R*^2^ values of all three models were higher than 0.5, showing that the urine dry chemical parameters better explained the variation in Ucol (more than 50%) and that Ucol was more influenced by the urine dry chemical parameters. This is an inspiring finding, as it further supports the strong relationship between urine dry chemical parameters and Ucol.

Finally, we determined the accuracy of the new classification method for hydration assessment (AUC = 0.892). The AUC of children was between 0.67 and 0.78 when using the eight-point color scale for self-assessing hydration (osmolality) (17, 22, 23). A similar AUC was found in a new (LUC) scale (osmolality.73 and USG.76) (34), suggesting that the results reported by the Ucol classification method with b^*^ were similar and even more accurate than those studies using the charts.

There are some limitations that should be mentioned. This cross-sectional study was conducted during the daily activities of the participants and the participants were not limited. The interruption of diet and medication on Ucol were not eliminated. In addition, previous studies (41) demonstrating that gender, age, and the collection time of urine samples (morning or non-morning) affected the result, and we did not bring in these factors. In terms of method, the digital colorimetric method has the advantage of being convenient and cheap, but its accuracy is limited. Although previous studies have successfully used this method for hydration and the trends are consistent, the color space values we measured were low and the equipment needs to be improved. In addition, the small and limited sample size only came from one university in China. Bedsides, relatively low normal samples and the universal differences between normal and abnormal urine dry chemistry parameters remain to be verified with larger sample sizes in the future.

Overall, this study demonstrated that grouping Ucol based on b^*^ value is an objective, simple, and practical method. At the same time, using digital imaging colorimetry to objectively quantify Ucol is a potential method to assess the hydration status of a person and, potentially, their health. Future studies should consider larger sampling and the variance of age, gender, and region to improve accuracy. The relationship between objective Ucol and other parameters, such as blood parameters, should also be examined and not limited to urine parameters. Furthermore, it should be combined with smart devices for application. At the same time, specific analyses should be performed on specific diseases in order to fit better in the clinic.

## Data Availability Statement

The raw data supporting the conclusions of this article will be made available by the authors, without undue reservation.

## Ethics Statement

The studies involving human participants were reviewed and approved by the Ethics Review Committee of Beijing University of Traditional Chinese Medicine (Approval Number: 2020BZYLL0301).

## Author Contributions

HZ, RZ, and WW conceived the study. JL and ZZ are the principal investigators of this trial. RZ, YC, and DM contributed to the collection of urine samples. ZZ and XH took pictures of the urine samples and extracted the data. JL and XP performed the data analysis. JL, ZZ, XP, and HZ drafted and revised the manuscript. All authors read and approved the final manuscript.

## Funding

This study was supported by the National Natural Science Foundation of China (No. 81973697).

## Conflict of Interest

The authors declare that the research was conducted in the absence of any commercial or financial relationships that could be construed as a potential conflict of interest.

## Publisher's Note

All claims expressed in this article are solely those of the authors and do not necessarily represent those of their affiliated organizations, or those of the publisher, the editors and the reviewers. Any product that may be evaluated in this article, or claim that may be made by its manufacturer, is not guaranteed or endorsed by the publisher.

## References

[1] Coppens A Speeckaert M Delanghe J. The pre-analytical challenges of routine urinalysis. Acta Clin Belg. (2010) 65:182–9. 10.1179/acb.2010.038 20669786

[2] Yu W Zhu S Ye C Zang L. Research progress of urine metabolites in tumor detection. J Hainan Med Univ. (2018) 24:1699–702.

[3] Echeverry G Hortin GL Rai AJ. Introduction to urinalysis: historical perspectives and clinical application. Methods Mol Biol. (2010) 641:1–12. 10.1007/978-1-60761-711-2_1 20407938

[4] Bouatra S Aziat F Mandal R Guo AC Wilson MR Knox C. The human urine metabolome. PLoS ONE. (2013) 8:e73076. 10.1371/journal.pone.0073076 24023812 PMC3762851

[5] Huan Y Wei J Su T Gao Y. Urine proteome changes in a chronic unpredictable mild stress (CUMS) mouse model of major depressive disorder. J Pharm Biomed Anal. (2021) 199:114064. 10.1016/j.jpba.2021.114064 33862505

[6] Ng K Stenzl A Sharma A Vasdev N. Urinary biomarkers in bladder cancer: a review of the current landscape and future directions. Urol Oncol. (2021) 39:41–51. 10.1016/j.urolonc.2020.08.016 32919875

[7] Brönimann S Pradere B Karakiewicz P Huebner NA Briganti A Shariat SF. An up-to-date catalogue of urinary markers for the management of prostate cancer. Curr Opin Urol. (2020) 30:684–8. 10.1097/MOU.0000000000000807 32701725

[8] Kouba E Wallen EM Pruthi RS. Uroscopy by hippocrates and theophilus: prognosis versus diagnosis. J Urol. (2007) 177:50–2. 10.1016/j.juro.2006.08.111 17161998

[9] Diamandopoulos AA. Uroscopy in byzantium. Am J Nephrol. (1997) 17:222–7. 10.1159/000169105 9189238

[10] Han X. Approaching the characteristic urinary diagnosis method of Tibetan medicine. Health News. (2019). 005.

[11] Yao X Xu S Bo X Luo Y. Overview of the research on the theoretical system of Tibetan medicine urinary diagnosis. Chin Natl Folk Med. (2013) 22:1–2.

[12] Liu J Tidwell T Zhao H Ren Q Mao M Wu H. Theoretical characteristics of Tibetan medicine. World J Tradit Chin Med. (2020) 6:490–9.

[13] Foot CL Fraser JF. Uroscopic rainbow: modern matula medicine. Postgrad Med J. (2006) 82:126–9. 10.1136/pgmj.2005.037598 16461475 PMC2596703

[14] Armstrong LE Maresh CM Castellani JW Bergeron MF Kenefick RW LaGasse KE. Urinary indices of hydration status. Int J Sport Nutr. (1994) 4:265–79. 10.1123/ijsn.4.3.265 7987361

[15] Armstrong LE Soto JA Hacker FT Jr Casa DJ Kavouras SA Maresh CM. Urinary indices during dehydration, exercise, and rehydration. Int J Sport Nutr. (1998) 8:345–55. 10.1123/ijsn.8.4.345 9841955

[16] Kavouras SA. Assessing hydration status. Curr Opin Clin Nutr Metab Care. (2002) 5:519–24. 10.1097/00075197-200209000-00010 12172475

[17] Perrier ET Bottin JH Vecchio M Lemetais G. Criterion values for urine-specific gravity and urine color representing adequate water intake in healthy adults. Eur J Clin Nutr. (2017) 71:561–3. 10.1038/ejcn.2016.269 28145416 PMC5383926

[18] Armstrong LE. Performing in Extreme Environments. Human Kinetics Press (1999).

[19] Perrier ET Johnson EC McKenzie AL Ellis LA Armstrong LE. Urine colour change as an indicator of change in daily water intake: a quantitative analysis. Eur J Nutr. (2016) 55:1943–9. 10.1007/s00394-015-1010-2 26286348 PMC4949298

[20] Hahn RG Waldréus N. An aggregate urine analysis tool to detect acute dehydration. Int J Sport Nutr Exerc Metab. (2013) 23:303–11. 10.1123/ijsnem.23.4.303 23994895

[21] Mentes JC Wakefield B Culp K. Use of a urine color chart to monitor hydration status in nursing home residents. Biol Res Nurs. (2006) 7:197–203. 10.1177/1099800405281607 16552947

[22] Kavouras SA Johnson EC Bougatsas D Arnaoutis G Panagiotakos DB Perrier E. Validation of a urine color scale for assessment of urine osmolality in healthy children. Eur J Nutr. (2016) 55:907–15. 10.1007/s00394-015-0905-2 25905541 PMC4819932

[23] Wardenaar FC Thompsett D Vento KA Pesek K Bacalzo D. Athletes' self-assessment of urine color using two color charts to determine urine concentration. Int J Environ Res Public Health. (2021) 18:4126. 10.3390/ijerph18084126 33924715 PMC8069841

[24] Choudhury A. Colour order systems. Coloration Technol. (2008) 26:54–62. 10.1111/j.1478-4408.1996.tb00110.x

[25] Chin J Tisan A. An IoT-based pervasive body hydration tracker (PHT). IEEE Int Conf Indust Inform. (2015) 437–41. 10.1109/INDIN.2015.7281774

[26] Chew N Azhar AMN Bustam A Azanan MS Wang C Lum LCS. Assessing dehydration status in dengue patients using urine colourimetry and mobile phone technology. PLoS Negl Trop Dis. (2020) 14:e0008562. 10.1371/journal.pntd.0008562 32881914 PMC7470395

[27] Huang JW Chen WC Huang TK Fu PS Lai PL Tsai CF. Using a spectrophotometric study of human gingival colour distribution to develop a shade guide. J Dent. (2011) 39:e11–6. 10.1016/j.jdent.2011.10.001 22005337

[28] Polo CG Montero J Casado AMM. Proposal for a gingival shade guide based on in vivo spectrophotometric measurements. J Adv Prosthodont. (2019) 11:239–46. 10.4047/jap.2019.11.5.239 31754413 PMC6856309

[29] Luo MR Cui G Rigg B. The development of the CIE 2000 colour-difference formula: CIEDE2000. Color Res Appl. (2001) 26:340–50. 10.1002/col.1049

[30] Zhang N Du S Zheng M Tang Z Yan R Zhu Y. Urine color for assessment of dehydration among college men students in Hebei, China - a cross-sectional study. Asia Pac J Clin Nutr. (2017) 26:788–93. 10.6133/apjcn.052017.09 28802286

[31] Edwards T Belasco R Munoz AJ Rayo V Buono M. Subjective vs. objective urine color: effect of hydration status. Adv Appl Physiol. (2020) 5:19–23. 10.11648/j.aap.20200502.12

[32] Belasco R Edwards T Munoz AJ Rayo V Buono MJ. The effect of hydration on urine color objectively evaluated in CIE L^*^a^*^b^*^ color space. Front Nutr. (2020) 7:576974. 10.3389/fnut.2020.576974 33195369 PMC7649145

[33] Sharma G Wu W Dalal EN. The CIEDE2000 color-difference formula: Implementation notes, supplementary test data, and mathematical observations. Color Res Appl. (2010) 30:21–30. 10.1002/col.20070

[34] Wardenaar FC Thompsett D Vento KA Bacalzo D. A lavatory urine color (LUC) chart method can identify hypohydration in a physically active population. Eur J Nutr. (2021) 60:2795–805. 10.1007/s00394-020-02460-5 33416980

[35] Liu G Yu J Ren L. Advances in RGB detection method. Chin J Anal Sci. (2020) 36:591–6.

[36] Yu Z Zhang M. The research progress of gingival colorimetry. J Dent Endod. (2018) 28:728–32.

[37] Armstrong LE. Assessing hydration status: the elusive gold standard. J Am Coll Nutr. (2007) 26(5 Suppl):575S−84S. 10.1080/07315724.2007.10719661 17921468

[38] Perrier ET Buendia-Jimenez I Vecchio M Armstrong LE Tack I Klein A. Twenty-four-hour urine osmolality as a physiological index of adequate water intake. Dis Markers. (2015) 2015:231063. 10.1155/2015/231063 25866433 PMC4381985

[39] Petejova N Martinek A Zadrazil J Teplan V. Acute toxic kidney injury. Ren Fail. (2019) 41:576–94. 10.1080/0886022X.2019.1628780 31237170 PMC6598532

[40] Yang Y. Research on color image quality evaluation based on spatial expansion of uniform color difference (Master. thesis). University of Science and Technology of China, China (2013).

[41] Ren Z. Basic theory of Tibetan medicine urine analysis and research on normal urine indexes (Master. thesis). Qinghai University, China (2013).

